# P-215. HIV/AIDS phobia, four decades into the epidemic: A cohort study in Venezuela

**DOI:** 10.1093/ofid/ofaf695.437

**Published:** 2026-01-11

**Authors:** David Forero-Peña, Lily Soto Avila, Oriana A Regalado-Gutiérrez, David Flora-Noda, Óscar Omaña-Ávila, Ivan Escalante-Pérez, Grecia Erimee, Napoleón Guevara, Martin Carballo, Andrea Maricuto, Natasha A Camejo-Avila, Carlos Morantes, Fhabian S Carrión-Nessi

**Affiliations:** Biomedical Research and Therapeutic Vaccines Institute, Ciudad Bolívar, Venezuela, Caracas, Distrito Federal, Venezuela; Infectious Diseases Department, Hospital Universitario de Caracas, Caracas, Venezuela, Caracas, Distrito Federal, Venezuela; Central University of Venezuela, Caracas, Miranda, Venezuela; Infectious Diseases Department, Hospital Universitario de Caracas, Caracas, Venezuela, Caracas, Distrito Federal, Venezuela; Universidad Central de Venezuela, Caracas, Miranda, Venezuela; “Luis Razetti” School of Medicine, Universidad Central de Venezuela, Caracas, Venezuela, Caracas, Distrito Federal, Venezuela; Universidad Central de Venezuela "Escuela de Medicina Luis Razetti", Caracas, Distrito Federal, Venezuela; Infectious Diseases Department, Hospital Universitario de Caracas, Caracas, Venezuela, Caracas, Distrito Federal, Venezuela; Universidad Central de Venezuela, Caracas, Miranda, Venezuela; Biomedical Research and Therapeutic Vaccines Institute, Ciudad Bolívar, Venezuela, Caracas, Distrito Federal, Venezuela; Universidad Central de Venezuela, Caracas, Miranda, Venezuela; Biomedical Research and Therapeutic Vaccines Institute, Ciudad Bolívar, Venezuela, Caracas, Distrito Federal, Venezuela; Biomedical Research and Therapeutic Vaccines Institute, Ciudad Bolívar, Venezuela, Caracas, Distrito Federal, Venezuela

## Abstract

**Background:**

HIV/AIDS phobia, defined as the persistent and irrational fear of contracting the infection besides contrary serologic evidence, was first described in 1983. Four decades into the epidemic and significant therapeutic advances, stigma and misinformation persist, potentially contributing to the perpetuation of this often-forgotten syndrome, whose diagnosis remains limited.

**Methods:**

This is a cohort study of patients who attended at Clinic of the University Hospital of Caracas and by direct contact with the infectologists of this institution through social media between May and September 2024, referring persistent fear of HIV infection after one or multiple risky sexual exposures.

**Results:**

A total of 17 patients were assessed, predominantly male (*n*=11, 64.7%), with a mean age of 31 years (SD ±6). Most were single (*n*=14, 77.8%), identifying as heterosexual (n=12, 66.7%) or homosexual (*n*=5, 27.8%), and a median of 1 (IQR 1-1) romantic partner and 2 (IQR 1-4) sexual partners in the last year. Most (*n*=11, 64.7%) had 1 to 4 risk exposures, primarily vaginal (*n*=13, 76.5%), oral (*n*=4, 23.5%), and active anal sex (*n*=3, 17.6%). Patients have visited a mean number of 3 doctors (SD ±1) and undergone 11 (SD ± 7) HIV diagnostic tests. They reported physical symptoms such as weight loss (*n*=10/17), odynophagia (*n*=10/17), and oral aphthae (*n*=7/17). Almost one-third (29.4%) received prescribed PEP, and 2 (11.8%) self-medicated with antiretroviral therapy (ART). Twelve (66.7%) patients underwent psychiatric assessment, presenting mainly with anxiety (*n*=17/17), insomnia (*n*=14/17), obsessive (*n*=10/17), and faulty ideas (*n*=8/17). Most common psychiatric diagnoses were illness anxiety disorder (45.5%) and somatic symptom disorder (27.3%). Patients had a median of 1 (IQR 1-3) psychiatry follow-up. However, none of them have shown a satisfactory progress.

**Conclusion:**

This result illustrates the significant, yet often overlooked, burden of HIV/AIDS phobia on affected individuals. The multiple unnecessary diagnostic tests and the indiscriminate use of ART point to a systemic failure in addressing this condition effectively. This syndrome urgently requires further research to determine its true prevalence and evaluate the effectiveness of appropriate therapeutic interventions.

**Disclosures:**

All Authors: No reported disclosures

